# The Role of Combination Antibiotic Therapy in Combatting Drug-Resistant *Acinetobacter baumannii* Infections: A Systematic Review of Randomised Control Trials

**DOI:** 10.3390/antibiotics15040356

**Published:** 2026-03-30

**Authors:** Anteneh Assefa Gezmu, Abel Workalemahu Tesfaye, Anthony R. M. Coates

**Affiliations:** 1 St George’s School of Health and Medical Sciences, City St George’s University of London, Cranmer Terrace, London SW17 0RE, UK; 2 School of Public Health, Yekatit 12 Hospital Medical College, Addis Ababa P.O. Box 257, Ethiopia; abelwmd@protonmail.com; 3 Medical Microbiology, St George’s School of Health and Medical Sciences, City St George’s University of London, Cranmer Terrace, London SW17 0RE, UK; 4 Helperby Therapeutics Group Ltd., 63 Bermondsey Street, London SE1 3XF, UK

**Keywords:** *Acinetobacter baumannii*, carbapenem-resistant *Acinetobacter baumannii*, antimicrobial resistance, randomized controlled trials, systematic review, combination antibiotic therapy, colistin-based therapy

## Abstract

Background: *Acinetobacter baumannii* is a major global health threat due to its rapid acquisition of multidrug resistance, particularly to carbapenems. Combination antibiotic therapy has been proposed to enhance antimicrobial activity and suppress resistance; however, evidence from randomized trials remains inconclusive. Methods: A systematic review of randomized controlled trials (RCTs) was conducted following PRISMA guidelines to evaluate the efficacy and safety of antibiotic combination therapy versus monotherapy for drug-resistant *A. baumannii* infections. Searches across MEDLINE, Embase, Global Health, and Cochrane Central (January 2010–June 2025) identified eligible RCTs reporting clinical outcomes. Data on clinical cure, mortality, microbiological eradication, adverse events, and resistance emergence are described narratively. Results: Eight RCTs enrolling 324 participants were included. Most trials investigated colistin-based combinations (e.g., colistin plus rifampicin, meropenem, fosfomycin, or sitafloxacin); one assessed tigecycline plus cefoperazone–sulbactam. No regimen demonstrated a significant mortality or clinical cure benefit over monotherapy, despite some combinations showing earlier or higher microbiological clearance, most notably colistin–fosfomycin and colistin–rifampicin, without corresponding improvement in clinical outcomes. Adverse events, predominantly nephrotoxicity, were common but comparable across groups. Heterogeneity in trial size, infection severity, and resistance mechanisms limited cross-study comparability. Conclusions: Current RCT evidence does not support routine use of combination therapy over monotherapy for drug-resistant *A. baumannii* infections, particularly in septic ICU populations where host factors dominate outcomes. Future trials should focus on early-stage or non-sepsis infections, incorporate molecular resistance profiling, and evaluate emerging agents such as sulbactam–durlobactam to guide precision therapy.

## 1. Introduction

### 1.1. Global Burden and Economic Impact of Antimicrobial Resistance

Antimicrobial resistance (AMR) is one of the greatest threats to global health, yet it remains underreported and underestimated. In 2019, AMR was directly responsible for an estimated 1.27 million deaths worldwide and associated with 4.85 million deaths, largely due to bacterial sepsis [1]. Without decisive action, the annual AMR-related mortality could rise to 10 million by 2050, surpassing cancer as a leading cause of mortality [2].

Beyond mortality, AMR imposes sustained pressure on health systems through prolonged hospitalisation, increased intensive care utilisation, and higher treatment costs. Resistant infections are consistently associated with longer lengths of stay and greater resource use across high-priority hospital pathogens, including *Acinetobacter baumannii* (hereafter, *A. baumannii*) [3]. These pressures extend into the wider economy. Syntheses of burden studies report substantial excess health expenditure and project losses to global GDP under high-resistance scenarios, underscoring how AMR steadily erodes service capacity [4]. Considered alongside the higher readmission risk reported by Poudel et al., 2023 [5], the mounting financial toll extends to the macroeconomy, where projections indicate up to US$1 trillion in additional healthcare costs annually by 2050 and US$1–3.4 trillion in GDP losses per year by 2030 (≈1.0–3.4% of world output), with the heaviest relative impacts in low- and middle-income countries (LMICs) [6,7]. Under high-burden AMR scenarios modelled by the World Bank and the O’Neill Review, cumulative global economic losses could exceed US$100 trillion by 2050, threatening to reverse decades of medical and economic progress [2,6].

### 1.2. Common Bacterial AMR as an Immediate Threat to Clinical Care

Although AMR affects diverse pathogen groups, resistance among common bacterial pathogens presents the most immediate threat to routine clinical care [1,7]. A key driver is horizontal gene transfer (HGT), through which plasmids, transposons and integrons facilitate rapid acquisition and dissemination of resistance determinants within and between species, accelerating the emergence and spread of multidrug-resistant (MDR), extensively drug-resistant (XDR) and pan drug-resistant (PDR) strains [8,9,10].

### 1.3. Disproportionate Impact of AMR in LOW and Middle Income Countries

The burden of AMR falls disproportionately on LMICs, where constrained laboratory capacity, limited antimicrobial stewardship, and fragile health systems amplify its impact [2,11]. This challenge is compounded by the coexistence of persistent infectious diseases and a rising burden of non-communicable conditions, stretching already limited healthcare resources [12,13]. In such settings, resistant infections frequently necessitate longer hospitalisation, more toxic or costly therapies, and are associated with poorer outcomes [13,14].

### 1.4. Acinetobacter Baumannii as a Critical Priority Pathogen

Within this context, carbapenem-resistant *Acinetobacter baumannii* (CRAB) has emerged as a critical priority pathogen. Recognised by the World Health Organization as a highest-priority organism for antibiotic development, *A. baumannii* is a prominent member of the ESKAPE group of nosocomial pathogens characterised by high levels of antimicrobial resistance and healthcare-associated transmission [15,16]. Its ability to persist in hospital environments, survive desiccation, and rapidly accumulate resistance mechanisms has rendered many first-line and last-resort agents, including carbapenems, increasingly ineffective [17,18,19].

### 1.5. Barriers to Antibiotic Development and Market Failure

Despite the urgent need for novel antibiotics, the global antibacterial development pipeline remains limited. Antibiotic research and development is costly and high risk, with failure-adjusted development costs approaching US$1 billion per successful agent, while stewardship-driven restricted use results in modest commercial returns [20,21,22]. These economic disincentives have contributed to widespread industry withdrawal from antibacterial R&D and repeated post-approval market failures, even for agents targeting WHO priority pathogens [23,24].

### 1.6. Combination Therapy as a Pragmatic Strategy Against Drug Resistance

In this therapeutic landscape, combination antibiotic therapy has been proposed as a pragmatic strategy to address infections caused by MDR pathogens. Combining agents with complementary mechanisms of action may enhance bacterial killing, suppress resistance emergence, and repurpose existing drugs more rapidly than de novo antibiotic discovery [25,26,27]. Such approaches are well established in tuberculosis and HIV management, but their role in CRAB infections remains uncertain, with concerns regarding toxicity, antagonism, and inconsistent clinical benefit [28].

### 1.7. Aim and Scope of This Review

This systematic review therefore critically evaluates the evidence from randomised controlled trials (RCTs) assessing combination antibiotic therapy for drug-resistant *Acinetobacter baumannii* infections, with a focus on clinical outcomes, microbiological efficacy, mortality, adverse events, and resistance emergence, to clarify the role of combination therapy in contemporary clinical practice.

## 2. Background: Epidemiology and Resistance Landscape of *Acinetobacter baumannii*

### 2.1. Epidemiology and Clinical Impact of A. baumannii

*Acinetobacter baumannii* is a Gram-negative, non-motile coccobacillus that has emerged as a major cause of healthcare-associated infections worldwide. Although *Acinetobacter* species are ubiquitous in the environment and can colonise human skin, *A. baumannii* is infrequently isolated from healthy individuals and is predominantly associated with hospital exposure, particularly in intensive care settings [18,29]. Its taxonomic status was clarified in the 1980s, when it was distinguished as a separate species from earlier groupings such as *Micrococcus calcoaceticus* [18,30].

Clinically, *A. baumannii* causes a wide spectrum of infections, most commonly ventilator-associated pneumonia (VAP), bloodstream infections (BSI), urinary tract infections (UTI), wound and surgical-site infections, and meningitis. The organism’s ability to survive prolonged desiccation and persist on hospital surfaces and medical devices facilitates transmission, particularly in high-dependency units and ICUs [17,18,31,32]. As a result, outbreaks are difficult to control once established.

Mortality associated with severe *A. baumannii* infections remains high, commonly ranging from 30% to 60%, especially among critically ill patients with pneumonia or bacteraemia [33,34,35]. Outcomes are strongly influenced by illness severity, comorbidities, and the timeliness and appropriateness of initial antimicrobial therapy, with delayed or ineffective treatment associated with markedly worse survival [36].

Carbapenem-resistant *A. baumannii* (CRAB) has expanded rapidly since initial reports in the late 1980s. In China, carbapenem resistance among clinical isolates increased from approximately 13% in 2004 to over 70% by 2018 [37]. Across Europe and the Eastern Mediterranean region, surveillance data indicate that many countries report carbapenem resistance rates exceeding 50%, with sustained increases through 2020–2021 [38,39]. In sub-Saharan Africa, pooled estimates suggest substantial resistance with marked between-country variation, likely compounded by under-reporting and limited laboratory capacity [11,40].

The clinical and economic consequences of CRAB infections are particularly severe in LMIC settings. Studies from India have demonstrated significantly higher per-patient costs for resistant bloodstream infections, with *A. baumannii* associated with one of the highest incremental cost burdens, driven largely by prolonged hospitalisation and antimicrobial expenditure [41]. Systematic reviews of Southeast Asian ICUs similarly identify *A. baumannii* as a major contributor to prolonged ICU stays and increased case fatality [42]. Comparable findings have been reported from African ICUs, where infections are associated with extended hospital stays and high mortality [43,44].

In high-income countries, carbapenem resistance in *Acinetobacter* species is also substantial, with 25 of 45 reporting countries in the WHO European Region documenting resistance rates of ≥50% in 2021 [45]. However, stronger infection prevention and control programmes, greater access to diagnostics, and more robust critical care infrastructure often mitigate the systemic impact compared with LMIC settings [39].

### 2.2. Mechanisms of Resistance in A. baumannii

Resistance in *A. baumannii* is multifactorial. Carbapenem resistance is driven mainly by class D OXA carbapenemases (for example OXA-23, OXA-24/40, OXA-58) and, less commonly, metallo-β-lactamases such as NDM, VIM or IMP. Fluoroquinolone resistance reflects gyrA/parC mutations with contributory RND efflux pumps (AdeABC/AdeIJK). Aminoglycoside resistance arises from modifying enzymes and 16S rRNA methylases (for example armA). Reduced susceptibility to polymyxins occurs through lipid A modification via pmrAB regulation or loss of lipooligosaccharide through lpxA/C/D disruptions. Together, these mechanisms underpin the frequent MDR and XDR phenotypes observed in clinical practice [18,46,47].

### 2.3. Classification of Drug Resistance and Clinical Relevance

Standardised definitions of resistance phenotypes are essential for surveillance, clinical communication, and research comparability. The consensus definitions proposed by Magiorakos et al. (2012) [48] remain widely adopted. Multidrug resistance (MDR) is defined as non-susceptibility to at least one agent in three or more antimicrobial categories; extensively drug resistance (XDR) denotes susceptibility to only one or two categories; and pan-drug resistance (PDR) reflects non-susceptibility to all agents tested [48].

While these categories are valuable for epidemiological reporting, they may misalign with clinical decision-making, particularly for non-fermenting Gram-negative pathogens such as *A. baumannii*. An isolate categorised as MDR or even XDR may remain treatable if activity is retained in a low-toxicity or high-efficacy agent, whereas another isolate classified similarly may have no viable therapeutic options [49].

To address this limitation, the “difficult-to-treat resistance” (DTR) framework was proposed to better capture clinical relevance. DTR is defined as non-susceptibility to all first-line, high-efficacy, low-toxicity β-lactams, including carbapenems and β-lactam/β-lactamase inhibitor combinations, as well as fluoroquinolones. This phenotype more accurately signals therapeutic dead ends and has been shown to correlate more strongly with adverse outcomes than MDR or XDR status alone [49,50]. Current Infectious Diseases Society of America (IDSA) guidance incorporates the DTR concept when addressing management of CRAB infections [51].

### 2.4. A Pragmatic Extension: XDR-Plus (XDR^+^)

Despite its utility, DTR does not fully capture challenges encountered in LMIC settings, where drug availability, toxicity, and access constraints substantially influence treatability. In recognition of this gap, this review adopts a pragmatic descriptor, XDR-plus (XDR^+^), to improve clinical signalling while retaining familiar terminology.

XDR^+^ denotes *A. baumannii* isolates that (i) meet XDR criteria and also fulfil a DTR-like profile, defined as non-susceptibility to all first-line, high-efficacy, low-toxicity β-lactams and fluoroquinolones, or (ii) meet XDR in vitro but, owing to local availability or toxicity constraints, have no accessible first-line therapeutic options. This designation is not intended to replace existing standards but rather to highlight scenarios in which treatment choices are effectively exhausted in real-world clinical practice, particularly in resource-limited settings [48,49,50,51].

### 2.5. Implications for Treatment Strategies and Rationale of This Review

Multiple therapeutic approaches have been investigated to manage carbapenem-resistant *A. baumannii* (CRAB), ranging from pathogen-targeted agents such as sulbactam–durlobactam and novel β-lactam/β-lactamase inhibitor combinations to newer antimicrobials like cefiderocol and polymyxin-based regimens [51,52]. Combination therapy, particularly those involving colistin or high-dose ampicillin–sulbactam backbones, has been widely explored in both clinical and in vitro settings.

However, the optimal role of these combination regimens remains debated, including whether they improve outcomes, limit resistance emergence or reduce toxicity compared with monotherapy. The evidence is heterogeneous, and treatment decisions often rely on limited randomised trial data, observational studies or in vitro synergy findings [51,53]. These uncertainties underpin the rationale for the present systematic review, which critically evaluates randomised controlled trial evidence on combination therapy for drug-resistant *A. baumannii* infections, focusing on clinical efficacy, microbiological outcomes, resistance emergence, and safety.

## 3. Results

### 3.1. Study Selection

**Figure 1:**
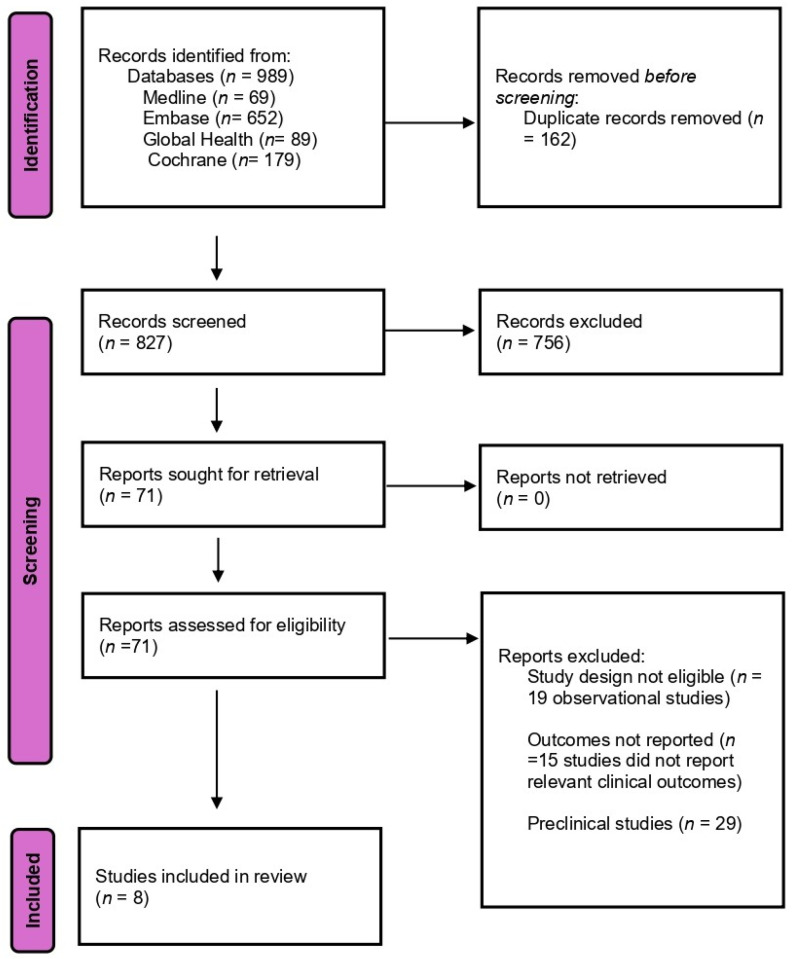
PRISMA flow diagram illustrating the identification, screening, eligibility assessment, and inclusion of randomized controlled trials evaluating combination antibiotic therapy versus monotherapy for drug-resistant *Acinetobacter baumannii* infections.

The database search identified 989 records. After de-duplication, 827 unique records underwent title–abstract screening; 71 full-text articles were assessed. The most common reasons for exclusion were ineligible design (observational; *n* = 19), lack of relevant clinical outcomes (*n* = 15), and preclinical studies (*n* = 29). Eight randomized controlled trials met the inclusion criteria (Figure 1).

### 3.2. Study Characteristics

Eight RCTs published between 2013 and 2022 were included, enrolling 1324 randomized participants across 10 countries (Table 1). Most trials were conducted in intensive care settings and focused on carbapenem-resistant or XDR *A. baumannii*. Sample sizes ranged from 9 [54] to 464 [55]. Seven trials evaluated colistin-based combinations, with rifampicin [54,56,57], meropenem [55,58], fosfomycin [59], and sitafloxacin [60]. One trial assessed tigecycline plus high-dose cefoperazone–sulbactam [61]. The only double-blind, placebo-controlled study was Kaye et al., 2022 (OVERCOME) [55], which compared colistin plus meropenem with colistin plus placebo. Outcomes typically included 28/30-day mortality, clinical response, microbiological eradication and adverse events, namely, nephrotoxicity. Primary outcomes and adverse events across studies are summarised in Table 2.

### 3.3. Risk of Bias

Risk of bias was assessed using RoB 2 across the five standard domains. One trial, Kaye et al., 2022 [55], was double-blind, placebo-controlled and judged low risk across all domains. In AIDA [58], randomization was robust, and outcome assessment was blinded, but the open-label intervention led us to judge some concerns for deviations from intended interventions; missing data and outcome measurement risks were low. Durante-Mangoni et al., 2013 [57], used central, stratified randomization with complete 30-day follow-up; open-label delivery again prompted some concerns for deviations from intended interventions, with other domains low risk. For the smaller, single-centre trials [54,56,59,60,61], randomization was reported but details of allocation concealment and blinded outcome assessment were limited; we therefore judged some concerns for the randomization and/or outcome-measurement domains, alongside some concerns for deviations from intended interventions inherent to open-label designs. Across studies, missing outcome data was low and generally balanced, and no clear evidence was found of selective reporting. Overall, seven trials were rated “some concerns,” and one [55] was rated “low risk”. Table 3 below summarizes these findings.

### 3.4. Clinical Outcomes

#### 3.4.1. Colistin Plus Rifampicin Versus Colistin Monotherapy

Three RCTs compared colistin plus rifampicin with colistin monotherapy in carbapenem-resistant *A. baumannii* infections [54,56,57]. Aydemir et al., 2013 [56] reported clinical cure rates of 45.5% for colistin versus 61.9% for combination therapy (RD = 16.5%, 95% CI −12.9 to 45.8, *p* = 0.290), which was not statistically significant, although time to microbiological clearance was shorter with combination therapy (3.1 ± 0.5 vs. 4.5 ± 1.7 days, *p* = 0.029). Durante-Mangoni et al., 2013 [57] found clinical cure in 60.6% of the combination arm versus 54.3% in monotherapy (RD = 5.7%, 95% CI −7.6 to 19.1, *p* = 0.43), again not statistically significant. Park et al., 2019 [54] reported clinical cure rates of 80.0% (colistin) versus 66.7% (colistin + rifampicin) (80.0% vs. 66.7%; *p* > 0.999).

#### 3.4.2. Colistin Plus Meropenem Versus Colistin Monotherapy

Two multicentre RCTs evaluated colistin plus meropenem versus colistin monotherapy [55,58]. Paul et al., 2018 [58] reported 14-day clinical failure rates of 82% in the colistin group versus 73% in the combination group (risk difference = −8.2%, 95% CI −18.8 to 2.4, *p* = 0.17). Kaye et al., 2022 [55] found no significant difference in clinical failure rates at the end of treatment between colistin plus meropenem and colistin alone (65% vs. 58%, RD = 6.8%, 95% CI −3.1 to 16.6, *p* = 0.17).

#### 3.4.3. Colistin Plus Fosfomycin Versus Colistin Monotherapy

Sirijatuphat & Thamlikitkul (2014) [59] reported clinical cure rates of 57.9% for colistin plus fosfomycin compared to 41.3% for colistin monotherapy (RD = 17.0%, 95% CI −2.9 to 36.9, *p* = 0.13), which was not statistically significant.

#### 3.4.4. Colistin Plus Sitafloxacin Versus Colistin Monotherapy

Sirijatuphat et al., 2022 [60] found end-of-treatment clinical cure rates of 81.5% with colistin plus sitafloxacin versus 77.8% with colistin alone (RD = 3.7%, 95% CI −17.8 to 25.2, *p* = 0.735), with no statistically significant difference.

#### 3.4.5. Tigecycline Plus Cefoperazone–Sulbactam Versus Tigecycline Monotherapy

Qin et al., 2018 [61] demonstrated significantly higher clinical effectiveness with tigecycline plus high-dose cefoperazone–sulbactam compared to tigecycline monotherapy (85.7% vs. 47.6%, *p* = 0.010).

**Figure 2:**
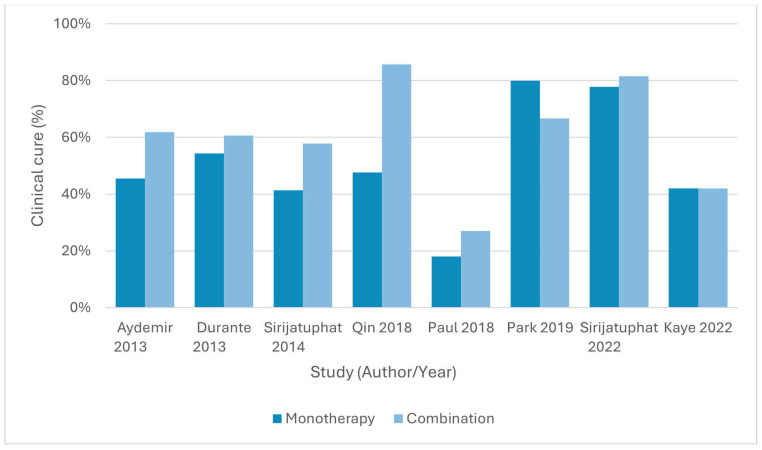
Clinical cure or clinical effectiveness rates in randomized controlled trials comparing combination antibiotic therapy with monotherapy for drug-resistant *Acinetobacter baumannii* infections. For trials reporting clinical failure, clinical cure was calculated as the complement of failure at the reported time point. No pooled estimates are shown [54,55,56,57,58,59,60,61].

Clinical cure or effectiveness rates across studies are presented in Figure 2.

### 3.5. Microbiological Outcomes

Seven RCTs reported microbiological eradication rates, although definitions and assessment time points varied across studies.

#### 3.5.1. Colistin Plus Rifampicin vs. Colistin Monotherapy

Aydemir et al., 2013 [56] reported mean time to microbiological clearance of 3.1 ± 0.5 days for combination therapy versus 4.5 ± 1.7 days for colistin monotherapy (*p* = 0.029), with final eradication rates of 71.4% versus 59.1% (RD = 12.3%, 95% CI −15.9 to 40.5; RR = 1.21, 95% CI 0.78–1.88, *p* = 0.597). Durante-Mangoni et al., 2013 [57] found significantly higher eradication rates in the combination group at day 7 (60.6% vs. 44.8%; RD = 15.2%, 95% CI 1.9 to 28.6; RR = 1.34, 95% CI 1.03–1.74, *p* = 0.034), but did not report microbiological eradication at the end of treatment. Park et al., 2019 [54], a small underpowered trial (*n* = 9), reported numerically higher day-14 clearance with the combination (100% vs. 40%, *p* = 0.196) but lacked statistical power to confirm significance.

#### 3.5.2. Colistin Plus Meropenem vs. Colistin Monotherapy

Paul et al., 2018 [58] reported no difference in microbiological failure rates between colistin plus meropenem and colistin monotherapy (31% vs. 35%; RR 1.10, 95% CI 0.84–1.44; *p* = 0.489). Kaye et al., 2022 [55] similarly found no meaningful differences in overall microbiologic cure rates (65% vs. 60%; RD = 4.8%, 95% CI −5.6 to 15.2), with no statistically significant difference between groups.

#### 3.5.3. Colistin Plus Fosfomycin vs. Colistin Monotherapy

Sirijatuphat & Thamlikitkul (2014) [59] reported significantly higher microbiological eradication with the combination at both 72 h (90.7% vs. 58.1%; RD = 32.6%, 95% CI 12.4 to 52.8; RR = 1.56, 95% CI 1.18–2.05, *p* = 0.001) and at end of treatment (100% vs. 81.2%, *p* = 0.01).

#### 3.5.4. Colistin Plus Sitafloxacin vs. Colistin Monotherapy

Sirijatuphat et al., 2022 [60] found no significant differences in eradication rates between the two groups, with end-of-treatment eradication of 73.1% versus 74.1% (RD = −3.7%, 95% CI −27.6 to 20.2; RR = 0.95, 95% CI 0.68–1.32, *p* = 0.934).

#### 3.5.5. Tigecycline Plus Cefoperazone–Sulbactam vs. Tigecycline Monotherapy

Qin et al., 2018 [61] did not report patient-level microbiological outcomes, focusing instead on clinical response and in vitro synergy testing.

Statistically significant and consistent microbiological benefit across time points was observed only with colistin plus fosfomycin [59]. Colistin plus rifampicin demonstrated earlier clearance [56] and higher eradication in one moderate-sized RCT [57], although results across studies, including the small Park et al., 2019 [54] trial, were inconsistent. Colistin plus meropenem [55,58] and colistin plus sitafloxacin [60] showed no significant microbiological advantage over monotherapy.

**Figure 3:**
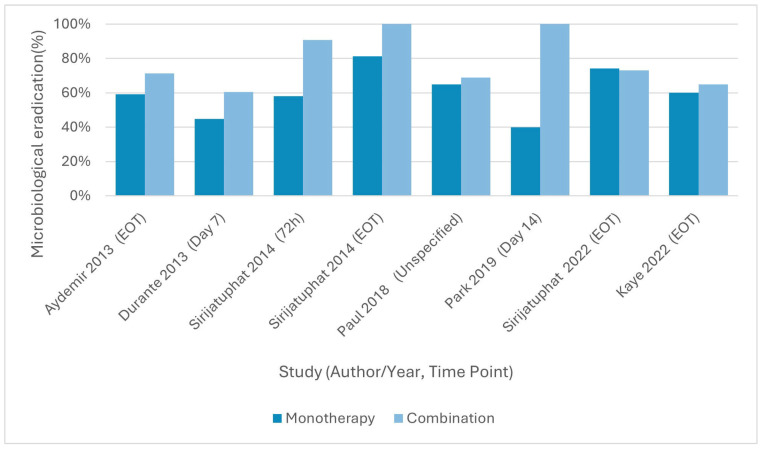
Microbiological eradication rates from randomized controlled trials evaluating combination therapy versus monotherapy for drug-resistant *Acinetobacter baumannii*. All trials reporting microbiological outcomes were included, with eradication shown at the time point reported in each study [e.g., day 7, day 14, or end of treatment (EOT)], as indicated under each author–year label. For studies reporting microbiological failure, eradication was calculated as 100% minus the failure rate. Results are presented descriptively without pooled estimates owing to differences in outcome definitions and timing across studies [54,55,56,57,58,59,60].

Microbiological eradication outcomes across studies are illustrated in Figure 3.

### 3.6. Mortality Outcomes

#### 3.6.1. Colistin Plus Rifampicin Versus Colistin Monotherapy

Aydemir et al., 2013 [56] reported VAP-related mortality of 38.1% in the colistin plus rifampicin group compared to 63.6% with colistin alone (RD = −25.5%, 95% CI −54.4 to 3.4; RR = 0.60, 95% CI 0.32–1.12; *p* = 0.171), with no fixed day specified. Durante-Mangoni et al., 2013 [57] assessed 30-day all-cause mortality, which was 43.3% for combination therapy versus 42.9% for monotherapy (RD = 1.0%, 95% CI −12.5 to 14.4; RR = 1.02, 95% CI 0.75–1.39; *p* = 0.95). Park et al., 2019 [54], a small underpowered trial (*n* = 9), reported 30-day mortality rates of 20.0% versus 33.3% in the combination and monotherapy groups, respectively (*p* = 1.000).

#### 3.6.2. Colistin Plus Meropenem Versus Colistin Monotherapy

Paul et al., 2018 [58] used 28-day mortality as a secondary outcome, reporting 52.0% mortality with colistin plus meropenem versus 46.0% with colistin alone (RD = 5.8%, 95% CI −4.0 to 15.5; RR = 1.12, 95% CI 0.92–1.37; *p* = 0.39). Kaye et al., 2022 [55] also evaluated 28-day mortality in the *A. baumannii* subgroup, reporting 42.0% for combination therapy versus 37.0% for monotherapy (RD = 5.0%, 95% CI −5.2 to 15.2; RR = 1.14, 95% CI 0.88–1.47; *p* = 0.17).

#### 3.6.3. Colistin Plus Fosfomycin Versus Colistin Monotherapy

Sirijatuphat & Thamlikitkul (2014) [59] reported 28-day all-cause mortality rates of 43.2% for combination therapy compared to 56.5% with colistin monotherapy (RD = −13.3%, 95% CI −34.7 to 8.1; RR = 0.76, 95% CI 0.51–1.15; *p* = 0.28).

#### 3.6.4. Colistin Plus Sitafloxacin Versus Colistin Monotherapy

Sirijatuphat et al., 2022 [60] found identical 28-day all-cause mortality rates for combination therapy and colistin monotherapy at 32.1% each (RD = 0.0%, 95% CI −24.4 to 24.4; RR = 1.00, 95% CI 0.61–1.63; *p* = 1.000).

#### 3.6.5. Tigecycline Plus Cefoperazone–Sulbactam Versus Tigecycline Monotherapy

Qin et al., 2018 [61] did not report mortality outcomes, focusing instead on clinical response rates and in vitro synergy testing.

**Figure 4:**
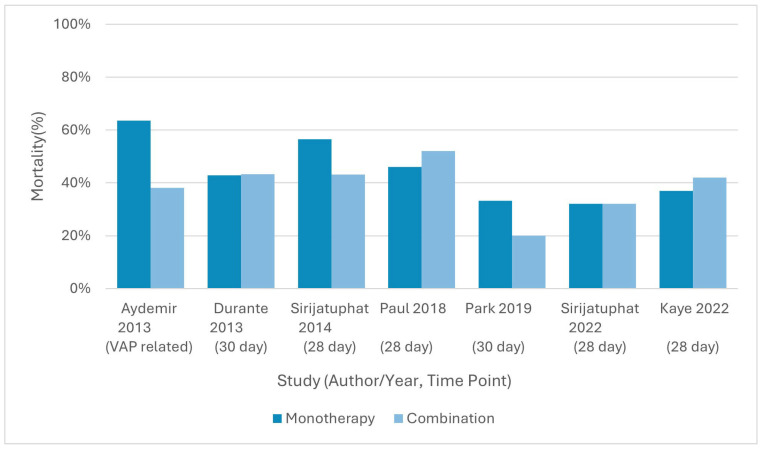
Mortality outcomes from randomized controlled trials evaluating combination therapy versus monotherapy for drug-resistant *Acinetobacter baumannii*. All trials reporting mortality were included, with mortality shown at the time point specified in each study (e.g., 28-day, 30-day, or infection-related), as indicated under each author–year label. Results are presented descriptively without pooled estimates owing to variation in outcome definitions and timing across studies [54,55,56,57,58,59,60].

Mortality outcomes across studies are presented in Figure 4.

### 3.7. Adverse Events

Adverse events were reported in seven of the eight RCTs, with nephrotoxicity consistently the most frequent complication. Hepatic, gastrointestinal, and neurological events were also reported across several studies.

#### 3.7.1. Colistin Plus Rifampicin vs. Colistin Monotherapy

Aydemir et al., 2013 [56] did not report per-arm nephrotoxicity data but noted no significant differences between groups. Durante-Mangoni et al., 2013 [57] reported renal dysfunction in approximately one quarter of patients overall (~26%), with no clear between group difference described, and no significant difference in hepatic dysfunction (20.8% [combination] vs. 11.9% [monotherapy]; *p* = 0.21).

#### 3.7.2. Colistin Plus Meropenem vs. Colistin Monotherapy

Paul et al., 2018 [58] reported renal failure at day 14 (RIFLE criteria) in 32.5% of the combination group versus 41.0% in the monotherapy group (RD = −8.5%, 95% CI −17.9 to 0.9; RR = 0.79, 95% CI 0.61–1.03; *p* = 0.26). Kaye et al., 2022 [55] found acute kidney injury in 49% versus 52% (RD = −3.4%, 95% CI −13.9 to 7.0; RR = 0.93, 95% CI 0.76–1.15; *p* = 0.55), with hypersensitivity reactions (1% vs. 3%; *p* = 0.22) and neurotoxicity (5% vs. 2%; *p* = 0.29) both rare and comparable between groups.

#### 3.7.3. Colistin Plus Fosfomycin vs. Colistin Monotherapy

Sirijatuphat & Thamlikitkul (2014) [59] reported acute kidney injury in 37.2% of the combination arm versus 48.7% of the monotherapy arm (RD = −11.5%, 95% CI −32.8 to 9.8; RR = 0.76, 95% CI 0.46–1.26; *p* = 0.28) and abnormal liver function tests in 11.6% versus 15.4% (*p* = 0.62).

#### 3.7.4. Colistin Plus Sitafloxacin vs. Colistin Monotherapy

Sirijatuphat et al., 2022 [60] found nephrotoxicity in 53.8% versus 45.8% (RD = 8.0%, 95% CI −19.6 to 35.7; RR = 1.17, 95% CI 0.67–2.06; *p* = 0.34), with electrolyte disturbances reported in both groups without significant differences.

#### 3.7.5. Tigecycline Plus Cefoperazone–Sulbactam vs. Tigecycline Monotherapy

Qin et al., 2018 [61] reported only mild gastrointestinal adverse events (e.g., nausea, diarrhoea) with no renal impairment in either arm.

Overall, no RCT demonstrated a statistically significant difference in overall adverse event profiles between combination therapy and monotherapy, although nephrotoxicity was common in all colistin-containing regimens.

**Figure 5:**
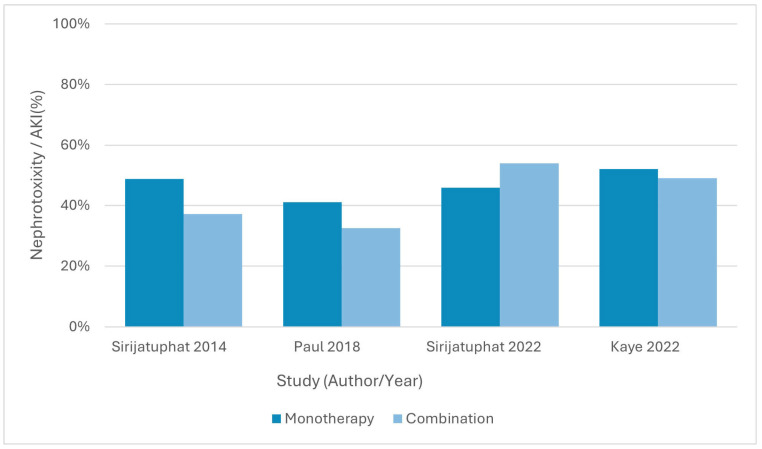
Nephrotoxicity or acute kidney injury reported in randomized controlled trials comparing combination antibiotic therapy with monotherapy for drug-resistant *Acinetobacter baumannii* infections. Only trials reporting per-arm renal toxicity outcomes were included. No pooled estimates are shown [55,58,59,60].

Rates of nephrotoxicity and acute kidney injury are summarised in Figure 5.

### 3.8. Development of Resistance

Emergence of resistance during therapy was inconsistently reported across the included RCTs. Two trials evaluating colistin–rifampicin combinations reported no development of colistin resistance in either arm, whereas rifampicin resistance was commonly reported among patients receiving combination therapy, with most affected individuals experiencing poor clinical outcomes [54,57]. In the AIDA trial, new colistin-resistant isolates were detected in 6% of patients receiving colistin monotherapy and 5% receiving colistin–meropenem, with no significant difference between groups [58]. A secondary analysis of the same trial confirmed these findings, reporting colistin resistance emergence in 9.4% versus 11.1% of monotherapy and combination therapy patients, respectively (*p* = 0.669), and demonstrating through molecular typing that most resistant strains were clonally related to baseline isolates, indicating resistance development during therapy rather than reinfection [62]. Other RCTs assessing colistin–fosfomycin, colistin–sitafloxacin, and tigecycline–cefoperazone–sulbactam did not report on-therapy resistance outcomes.

## 4. Discussion

### 4.1. Summary of Main Findings

This systematic review of randomized controlled trials found no consistent mortality or clinical cure benefit from adding a second antibiotic to treat drug-resistant *Acinetobacter baumannii*, most commonly carbapenem-resistant strains (CRAB) [54,55,56,57,58,59,60]. Across trials, absolute risk differences (RDs) for mortality ranged from −25.5% to +5.8%, with most 95% confidence intervals crossing zero, indicating substantial uncertainty and no clear survival benefit. Similarly, clinical cure outcomes were generally comparable between combination therapy and monotherapy; only a small single-centre trial, Qin et al., 2018 [61], demonstrated a statistically significant improvement, which was not replicated in larger multicentre RCTs [55,58].

Several trials observed earlier or higher microbiological clearance with combination therapy. For instance, in ventilator-associated pneumonia due to CRAB, adding rifampicin reduced the mean time to culture negativity from 4.5 to 3.1 days (*p* = 0.029), yet VAP-related mortality remained 38.1% vs. 63.6% (RD = −25.5%, 95% CI −54.4 to 3.4), showing no definitive survival benefit [56]. Likewise, colistin–fosfomycin achieved significantly higher eradication rates at 72 h (RD = 32.6%, 95% CI 12.4 to 52.8) and at end of therapy (100% vs. 81.2%; *p* = 0.01) without improving clinical outcomes [59].

One non-colistin combination trial, tigecycline plus high-dose cefoperazone–sulbactam, showed significantly higher clinical effectiveness compared to tigecycline alone (85.7% vs. 47.6%; *p* = 0.010), but mortality and microbiological outcomes were not reported [61].

Collectively, these RCT findings demonstrate that microbiological success does not reliably translate into clinical cure or survival benefit, underscoring the persistent disconnect between pathogen eradication and patient-centred outcomes.

These results are corroborated by recent meta-analytic findings. A rapid systematic review using Bayesian meta-analysis concluded that, for CRAB infections, colistin–meropenem offers no superiority over colistin monotherapy in clinical outcomes [63]. A comprehensive meta-analysis pooling RCTs and observational data similarly found comparable clinical improvement and mortality between colistin monotherapy and colistin–meropenem, although combination therapy showed a modest microbiological benefit [64]. Commentary on this analysis emphasised study heterogeneity and reinforced that RCT-focused evidence shows no clinical advantage for colistin-based combinations [64]. This consensus is increasingly reflected in expert guidance, which does not support the specific colistin-based combinations evaluated in randomised trials, particularly colistin–meropenem, and cautions against adjunctive agents such as rifampicin or fosfomycin in the context of colistin-based regimens due to lack of clinical benefit [65,66].

### 4.2. Lack of Mortality Benefit in Sepsis: Pathophysiological Drivers and Timing

The lack of mortality benefit in the included trials can be explained by the complex pathophysiology of sepsis and the critical importance of antibiotic timing. Sepsis triggers a dysregulated immune response characterised by an initial hyperinflammatory phase leading to systemic inflammatory response syndrome (SIRS), microcirculatory dysfunction, and progressive multi-organ failure, followed by a state of immunosuppression that persists even after bacterial clearance [67,68,69,70]. By the time antibiotic therapy is initiated, often when organ injury is already advanced, eliminating the pathogen may not reverse these processes [67,69].

Timeliness of therapy is therefore critical. Observational studies demonstrate that each hour’s delay in administering appropriate antibiotics increases mortality; adjusted odds increased by ~9% per hour in sepsis, rising to an absolute 1.8% mortality increase in septic shock [68]. Earlier research similarly found a 7.6% decrease in survival per hour in septic shock when antibiotics were delayed [71], and a contemporary multicentre study showed a 35% increased mortality risk per hour delay within 3 h among septic shock patients [72].

This principle is supported by the two largest multicentre RCTs in this review, AIDA [58] and OVERCOME [55], which enrolled critically ill patients with pneumonia, bacteraemia, or septic shock. Many required mechanical ventilation, vasopressors, or renal replacement therapy, indicating severe baseline illness. In both trials there was no difference in 28-day mortality between combination therapy and colistin monotherapy: AIDA RD = +5.8% (95% CI −4.0 to 15.5) and OVERCOME RD = +5.0% (95% CI −5.2 to 15.2). Given this advanced stage and delayed therapy initiation, combination antibiotics were unlikely to alter outcome trajectories. Although these mechanisms plausibly explain the lack of observed benefit, residual confounding related to illness severity, timing of therapy, and source control cannot be fully excluded.

The smaller single-centre RCTs, Aydemir et al., 2013 [56], Durante-Mangoni et al., 2013 [57], Sirijatuphat & Thamlikitkul (2014) [59], Sirijatuphat et al., 2022 [60], also reported no mortality differences, with effect sizes again consistent with no clear survival benefit: Aydemir RD = −25.5% (95% CI −54.4 to 3.4); Durante-Mangoni RD = +1.0% (95% CI −12.5 to 14.4); Sirijatuphat 2014 RD = −13.3% (95% CI −34.7 to 8.1); Sirijatuphat 2022 RD = 0.0% (95% CI −24.4 to 24.4). Qin et al., 2018 [61] did not report mortality outcomes. These studies also lacked granular data on illness severity, timing, or source control measures, limiting further insights into why outcomes remained unchanged.

Moreover, sepsis-induced immune dysregulation extends beyond the initial inflammatory storm. Immunoparalysis, characterised by monocyte dysfunction, apoptosis of immune effectors, and impaired pathogen clearance, can persist well into illness, undermining recovery and survival even when bacterial eradication is achieved [68,73,74]. Collectively, these findings align with the consensus that mortality in septic shock is primarily driven by host-mediated organ dysfunction rather than persistent infection alone [67,69].

### 4.3. Microbiological Response vs. Clinical Outcomes

Across the eight included RCTs, combination regimens often produced earlier or higher microbiological eradication rates but failed to improve clinical cure or survival outcomes [54,55,56,57,58,59,60,61]. For example, in ventilator-associated pneumonia, colistin–rifampicin significantly shortened the time to sputum sterilisation compared with colistin monotherapy (3.1 ± 0.5 vs. 4.5 ± 1.7 days; *p* = 0.029) and final eradication was 71.4% vs. 59.1% (RD = 12.3%, 95% CI −15.9 to 40.5; RR = 1.21, 95% CI 0.78–1.88) without affecting mortality [56]. Similarly, in bloodstream infections, rifampicin–colistin achieved faster bacteriological clearance, with day-7 eradication 60.6% vs. 44.8% (RD = 15.2%, 95% CI 1.9 to 28.6; RR = 1.34, 95% CI 1.03–1.74), yet 30-day mortality remained unchanged [57]. In a small, underpowered trial, colistin–rifampicin showed higher day-14 clearance (100% vs. 40%), but with wide confidence intervals and no mortality benefit [54]. This dissociation between microbiological clearance and clinical outcomes has been observed in other severe bacterial infections and reflects the complex interaction between pathogen eradication and host response. While faster bacterial clearance may reduce microbial burden, it does not necessarily reverse the inflammatory cascade that characterizes established sepsis and organ dysfunction [75,76]. The included RCTs did not assess immunological markers, cytokine profiles, or trajectories of organ dysfunction, making it difficult to determine whether improved microbiological outcomes translate into meaningful clinical recovery. Consequently, the mechanisms underlying this disconnect remain biologically plausible but cannot be directly confirmed from the available trial data.

This discrepancy reflects the underlying pathophysiology of sepsis. Once systemic inflammation and multi-organ dysfunction are established, pathogen eradication alone rarely reverses host-mediated injury. Consequently, patients often die from refractory septic shock and organ failure rather than uncontrolled microbial replication itself [67,69].

Importantly, one RCT focusing on ventilator-associated pneumonia did not report patient-level microbiological eradication outcomes, limiting cross-trial comparability and reinforcing the need for standardised endpoints encompassing both clinical and microbiological parameters [61]. By contrast, colistin–meropenem trials showed no microbiological advantage. In AIDA [58], microbiological failure was 31% with combination therapy versus 35% with colistin alone (RR = 1.10, 95% CI 0.84–1.44), and in OVERCOME [55], microbiologic cure was 65% versus 60% (RD = 4.8%, 95% CI −5.6 to 15.2).

Overall, while bacterial eradication remains a key therapeutic target, it alone is insufficient to improve survival in critically ill patients with *A. baumannii* infections [55,58]. Future RCTs should adopt composite endpoints and evaluate interventions at earlier stages of infection before irreversible organ injury occurs [67,69].

### 4.4. Infection Severity and Trial Context: Sepsis vs. cUTI

The contrasting results between CRAB sepsis trials and studies in less acute infections such as complicated urinary tract infections (cUTIs) highlight fundamental differences in patient populations and trial design. In cUTI, patients are generally haemodynamically stable with infections confined to the urinary tract, reducing the risk of severe systemic complications. This clinical stability enables antibiotic efficacy to be assessed in a controlled setting with fewer confounders such as multi-organ failure, septic shock, or competing causes of death [75].

The ASPECT-cUTI phase III trial, for instance, compared ceftolozane–tazobactam with high-dose levofloxacin and reported significantly higher composite clinical and microbiological cure for ceftolozane–tazobactam (76.9% vs. 68.4%; RD = 8.5 percentage points, 95% CI 2.3–14.6) [75]. Such trials benefit from well-defined endpoints, longer follow-up periods, and relatively homogeneous patient groups, enhancing statistical power and interpretability [77].

In contrast, the CRAB RCTs included in this review enrolled critically ill ICU patients with severe infections such as pneumonia, bacteraemia, or septic shock [54,55,56,57,58,59,60,61]. In these populations, outcomes such as mortality are strongly influenced by host-related factors like cytokine storm, comorbidities, and multi-organ dysfunction, as well as by non-antibiotic interventions including source control, mechanical ventilation, and vasopressor use [67,69]. Reflecting this, the two largest multicentre trials, AIDA and OVERCOME, showed no mortality benefit for adding meropenem to colistin (AIDA RD = +5.8%, 95% CI −4.0 to 15.5; OVERCOME RD = +5.0%, 95% CI −5.2 to 15.2), underscoring how severity and competing risks can obscure antibiotic efficacy signals [55,58].

These differences explain why antibiotic efficacy signals frequently emerge in cUTI trials but remain obscured in sepsis RCTs, where infection severity and systemic complications dominate outcomes regardless of antimicrobial regimen [55,58,75].

### 4.5. Limitations of Trials and Evidence Gaps

This review highlights significant limitations in the existing RCT evidence on combination therapy for drug-resistant *A. baumannii*. Only eight trials met inclusion criteria, many with small sample sizes; several enrolled fewer than 50 patients per arm, limiting statistical power to detect meaningful differences [54,55,56,57,58,59,60,61]. The difficulty of recruiting sufficient patients with multidrug-resistant infections, which are often sporadic, outbreak-related, and concentrated in ICUs, led to early withdrawals and incomplete follow-up in several trials [55,57,58]. This raises the risk of type II error, where real treatment effects may be missed due to underpowered designs [76].

Substantial heterogeneity in trial design, patient populations, and outcome definitions further complicates interpretation. Infection sources ranged from pneumonia and bloodstream infections to urinary tract infections and mixed foci, enrolling patients with markedly different prognoses [56,58,59,61]. Some studies defined endpoints as binary outcomes (e.g., clinical cure or mortality), whereas others used composite clinical failure measures incorporating physiological and laboratory parameters [57,58]. Follow-up durations also varied widely, from 14 to 30 days [58,59], reducing comparability across trials.

Most studies were open-label rather than double-blind, introducing risk of bias in patient management and outcome assessment [55,57,58]. While mortality represents an objective endpoint, clinical cure assessments may be influenced by investigator judgement and knowledge of treatment allocation. Consequently, subjective outcomes such as clinical improvement or cure may be more susceptible to assessment bias in open-label studies. Furthermore, the frequent use of rescue or crossover therapies in deteriorating patients likely diluted any differences between study arms [55,58].

Geographical variation adds another layer of complexity. Trials conducted in Asia frequently involved *OXA-type carbapenemase*-producing *A. baumannii*, while European studies more often reported *metallo-β-lactamase* or mixed mechanisms [55,58,77]. Where carbapenemase production is universal, adding a carbapenem antibiotic is unlikely to provide benefit, potentially explaining negative findings in some trials [57,58]. Conversely, signals of improved bacterial clearance with colistin–fosfomycin in Thai studies may reflect regional susceptibility patterns rather than universally applicable effects [59]. Moreover, heterogeneity extended to antibiotic regimens themselves, with one tigecycline-based combination trial [61] contrasting with predominantly colistin-based studies, further complicating cross-trial comparisons.

Collectively, these limitations restrict the external validity of findings and underscore the urgent need for large, multicentre, double-blind RCTs with standardised endpoints, stratification by infection source, and molecular resistance profiling [55,58].

### 4.6. Economic and Regulatory Barriers to New Antibiotics

The lack of effective therapies for CRAB is rooted in entrenched economic, regulatory, and scientific disincentives that have severely restrained antibiotic innovation. Antibiotics are far less attractive to pharmaceutical companies compared to chronic disease drugs or biologics since they involve short treatment durations, are reserved as last-resort agents, and are deliberately used sparingly to preserve efficacy [78,79]. A typical development cost for a new antibiotic reaches approximately US$1.2 billion, while global sales are often capped below US$100 million annually, yielding a dismal return on investment [79]. Consequently, many major pharmaceutical firms have withdrawn from antibiotic R&D, shifting the burden to smaller, nonprofit entities [78].

This untenable situation, where public health expects rapid antibiotic development yet enforces restrictive use, has rendered the market for antimicrobials effectively “broken” [78]. Regulatory barriers further exacerbate the problem; as demonstrating clinical superiority in lethal infections is practically impossible, ethical concerns prevent placebo use, and trials are often limited to small, non-inferiority designs with inconsistent endpoints [79].

To address these issues, several global mechanisms are emerging. CARB-X offers “push” funding for early-stage antibiotic R&D, supporting vaccines, diagnostics, and therapies [80]. GARDP is another nonprofit initiative jointly created by WHO targeting late-stage R&D and equitable access [81]. The UK has piloted a “Netflix-style” subscription model, providing a fixed annual fee for access to new antibiotics regardless of usage, to delink revenue from volume and safeguard innovation [82,83]. In the US, the proposed PASTEUR Act aims to further these pull incentives by offering substantial rewards post-approval to sustain antibiotic availability [84].

Despite these efforts, global alignment remains fragmented, and many proposals still lack financing models that sustain the large-scale clinical trials needed for new antibiotic approval. Without sustainable economic incentives, the pipeline for novel agents against CRAB and similar threats will remain dangerously fragile [78,79].

### 4.7. Future Directions and Novel Therapies

Given the limited efficacy of existing antibiotic combinations, future strategies must embrace novel therapeutics, precision medicine, and adjunctive interventions. Among the most promising developments is the novel β-lactam/β-lactamase inhibitor combination sulbactam–durlobactam (SUL-DUR), specifically designed for *A. baumannii*. SUL-DUR restores potency against *A. baumannii* by inhibiting class A, C, and D β-lactamases, with preclinical studies reporting low resistance rates (~2.3%) among carbapenem-resistant isolates [85]. Phase 1 studies demonstrated favourable pharmacokinetics and safety [86]. In the phase 3 ATTACK trial, SUL-DUR combined with imipenem was non-inferior to colistin plus imipenem for 28-day mortality (19% vs. 32%; difference −13.2%, 95% CI −30.0 to 3.5) and significantly reduced nephrotoxicity (13% vs. 38%, *p* < 0.001). This represents a paradigm shift toward pathogen-targeted therapies, aligning treatment strategies with molecular resistance mechanisms rather than empiric escalation alone [24].

Other agents under evaluation include Cefiderocol, a siderophore–cephalosporin exploiting bacterial iron uptake pathways to penetrate resistant Gram-negative pathogens [87]. Although Cefiderocol exhibits potent in vitro activity against CRAB, clinical outcomes remain mixed, with some studies noting higher mortality compared to best available therapy [87]. Combination regimens and earlier-stage use are under investigation to optimise efficacy [88]. The emergence of targeted agents such as sulbactam–durlobactam and cefiderocol may also influence the future role of combination antibiotic therapy in CRAB infections. Historically, colistin-based combinations were often used empirically to enhance activity against highly resistant pathogens. In contrast, newer agents are designed to directly overcome key resistance mechanisms, including class D β-lactamases or through siderophore-mediated bacterial uptake pathways [85,86,87]. As these pathogen-directed therapies become more widely available, the routine use of broad empirical combination regimens may become less necessary. Future randomized trials should therefore evaluate not only combination strategies but also the optimal positioning of these newer agents within treatment algorithms guided by molecular resistance profiles.

Beyond small-molecule drugs, non-traditional therapies are attracting interest. Bacteriophage therapy has shown encouraging results in case reports and early-phase trials for multidrug-resistant *A. baumannii* infections, particularly where conventional therapies have failed [89]. Monoclonal antibodies targeting virulence factors, such as the outer membrane protein Omp38, have shown promise in preclinical models by inhibiting biofilm formation and bacterial adherence [90]. Other antibody candidates (e.g., mAb 8E6 and mAb 1B5) demonstrated broad targeting of MDR *A. baumannii* strains and enhanced bactericidal activity in mice [91]. Additionally, a capsular polysaccharide-specific mAb (mAb1416) provided prophylactic protection in murine models of carbapenem-resistant *A. baumannii* infection [92].

Vaccine strategies targeting conserved antigens, such as OmpW, FilF, BamA, and outer membrane vesicle components, have shown immunogenicity and protective effects in animal studies but remain in preclinical phases [93,94].

Finally, integrating precision diagnostics, including rapid molecular resistance panels and host-response biomarkers, has the potential to facilitate real-time tailoring of therapy based on infection severity and resistance genotype. These tools may enable earlier initiation of effective therapy while preserving antibiotic efficacy through targeted use, thereby supporting antimicrobial stewardship globally [95,96,97].

### 4.8. Global Health Implications

Drug-resistant *A. baumannii* represents a critical global health threat, disproportionately affecting LMICs where ICU capacity, infection prevention infrastructure, and access to novel antimicrobials are often limited [95]. Antimicrobial resistance (AMR) is already estimated to contribute to nearly 5 million deaths annually and could account for 10 million deaths per year by 2050 without urgent intervention [1,2].

The lack of mortality benefit from existing antibiotic combinations underscores the need for comprehensive strategies beyond drug development alone. While novel agents such as sulbactam–durlobactam and cefiderocol represent important therapeutic advances, equitable global access remains a major barrier. Without mechanisms such as tiered pricing, global pooled procurement, or public–private partnerships, many high-burden regions may face years of delay before accessing life-saving treatments [95].

Strengthening health system capacity in LMICs is equally essential. This includes improving diagnostic infrastructure for rapid resistance detection, expanding antimicrobial stewardship programmes to preserve the efficacy of new drugs, and investing in infection prevention and control (IPC) measures to reduce transmission in hospitals [45].

Finally, the global threat of CRAB highlights the need for a One Health approach linking human, animal, and environmental health sectors to limit antimicrobial misuse across domains [98]. The convergence of drug resistance, health system weaknesses, and inequitable access demands coordinated international policies to prevent widening disparities in infectious disease outcomes.

### 4.9. Strengths and Limitations of This Review

A key strength of this review lies in its exclusive focus on eight randomised controlled trials (RCTs), representing the highest level of evidence for evaluating combination antibiotic therapy in *A. baumannii* infections [54,55,56,57,58,59,60,61]. Restricting inclusion to RCTs minimises the confounding and selection biases inherent in observational studies, providing a more robust assessment of therapeutic efficacy. The comprehensive search strategy across multiple databases, supplemented by manual reference screening and guided by PRISMA recommendations, further strengthens methodological rigour [99,100,101].

However, several limitations must be acknowledged. The small number of eligible RCTs, each with modest sample sizes, limits statistical power to detect clinically meaningful differences. Heterogeneity in infection sources, resistance mechanisms, treatment regimens, and outcome definitions limited comparability across studies and precluded meta-analysis. The restriction to English-language publications may have excluded trials conducted in high-burden regions where *A. baumannii* is endemic.

Methodologically, six of the eight RCTs used open-label designs rather than double-blinding, introducing potential performance and detection bias. Furthermore, key effect modifiers, such as timing of antibiotic initiation, adequacy of source control, and local resistance epidemiology, were inconsistently reported across most studies, limiting the generalisability of findings beyond the included settings.

Despite these constraints, this review provides the most comprehensive synthesis of RCT evidence to date, underscoring the need for adequately powered, methodologically rigorous trials to inform optimal management of multidrug-resistant *A. baumannii* infections.

## 5. Materials and Methods

### 5.1. Study Design and Search Strategy

This systematic review was conducted in accordance with the Preferred Reporting Items for Systematic Reviews and Meta-Analyses (PRISMA) guidelines [102].

A review protocol was developed prior to study initiation to guide eligibility criteria, data extraction, and analysis; however, the protocol was not prospectively registered in a public registry (e.g., PROSPERO). The aim was to evaluate the efficacy of antibiotic combination therapies for drug-resistant *A. baumannii* infections.

A comprehensive electronic search was performed using the Ovid platform to access MEDLINE, Embase, and Global Health databases, alongside the Cochrane Central Register of Controlled Trials (CENTRAL). To ensure completeness, reference lists of all eligible studies were manually screened for additional relevant publications [101]. The final search was conducted on 13 June 2025.

The search included studies published between January 2010 and June 2025. This time frame was selected because foundational clinical and in vitro studies assessing synergistic effects of antibiotic combinations began to emerge around 2010 [103,104], preceding the designation of *A. baumannii* as a WHO critical priority pathogen in 2017 [16]. Restricting the search to this period ensured capture of all key studies informing the development and evaluation of combination therapies.

Only randomized controlled trials (RCTs) were included, as they represent the highest standard of clinical evidence for treatment efficacy [105,106]. Observational studies, case reports, and reviews were excluded.

Both Medical Subject Headings (MeSH) and free-text keywords relating to *A. baumannii*, antimicrobial resistance, and combination therapy were used. The full electronic Boolean search strategies for all databases are provided in Supplementary Table S1.

Meta-analysis was not performed due to substantial heterogeneity in interventions, patient populations, and outcome measures across studies. Instead, a structured narrative synthesis was conducted to allow contextual interpretation of the evidence.

### 5.2. Eligibility Criteria

Studies were eligible if they were peer-reviewed, full-text RCTs comparing combination antibiotic therapy with monotherapy for the treatment of confirmed drug-resistant *A. baumannii* infections in human subjects.

A modified PICO framework was used to guide the eligibility criteria. The population included patients with infections caused by MDR, XDR, or XDR-plus *A. baumannii*. The intervention of interest was systemic combination antibiotic therapy, compared against systemic monotherapy. Eligible studies were required to report at least one clinical outcome, such as clinical cure, clinical improvement, or 28-/30-day all-cause or infection-related mortality.

Studies reporting additional outcomes, including microbiological eradication, resistance development, or adverse events, were included only if clinical outcomes were also presented. Mixed-pathogen RCTs were eligible when *A. baumannii* subgroup outcomes were extractable; only *A. baumannii*-specific results were synthesised.

Only English-language studies published from January 2010 onward were eligible. Excluded studies included observational studies, preclinical studies, case reports, conference abstracts, reviews, studies lacking full-text access, or those evaluating non-antibiotic interventions. The final inclusion and exclusion criteria are summarized in Table 4.

### 5.3. Screening

All search results were imported into Rayyan (Rayyan Systems Inc., Cambridge, MA, USA) for duplicate removal and title–abstract screening, while Zotero (Version 7.0.32, Corporation for Digital Scholarship, Vienna, VA, USA) was used for reference management. Screening was conducted in two stages: first, titles and abstracts were independently screened by two reviewers using predefined eligibility criteria; second, full-text screening was performed for all potentially eligible studies, with reasons for exclusion documented. Discrepancies were resolved through discussion until consensus was reached, with a third reviewer available for arbitration if required, though this was not necessary. The PRISMA flow diagram (Figure 1) summarizes the selection process, including the number of records identified, screened, assessed for eligibility, and included in the final synthesis.

### 5.4. Data Extraction

Data from all included studies were extracted into a standardized Microsoft Excel (Microsoft Corporation, Redmond, WA, USA) form developed for this review. The following variables were collected:

- Study characteristics: First author, year of publication, country, journal, single- or multi-centre design.
- Participant details: Inclusion criteria, number of patients, age, sex, comorbidities, infection type (e.g., ventilator-associated pneumonia, bloodstream infection), *A. baumannii* resistance classification (MDR, XDR, CRAB, XDR-plus).
- Intervention and comparator details: Antibiotics used in combination therapy (drug names, doses, administration routes, frequency, duration) and monotherapy regimen details.
- Clinical outcomes: Clinical cure or improvement, all-cause or infection-related mortality at 28 or 30 days.
- Additional outcomes (if reported):
  1. Microbiological eradication (e.g., negative cultures at end of therapy).
  2. Development of resistance during treatment (e.g., emergence of resistant strains, changes in MIC), including detection methods, timing, affected antibiotics, and differences between treatment arms.
  3. Adverse events or treatment-related toxicities.

All extracted data were independently checked by two reviewers prior to synthesis.

Clinical outcomes referred to patient-centred endpoints such as clinical cure, improvement in infection-related signs and symptoms, or mortality. Microbiological outcomes referred to eradication of *Acinetobacter baumannii* from clinical cultures during or after therapy.

### 5.5. Risk of Bias Assessment

The methodological quality of all included randomized controlled trials was assessed using the Cochrane Risk of Bias 2 (RoB 2) tool [107], which evaluates bias across five domains: the randomization process, deviations from intended interventions, missing outcome data, measurement of the outcome, and selection of the reported result. Each domain was rated as having low risk, some concerns, or high risk of bias. Two reviewers independently conducted the assessments, and discrepancies were resolved through discussion until consensus was reached.

### 5.6. Statistical Analysis and Data Synthesis

Dichotomous outcomes were summarized using proportions and effect estimates as reported by the original trial authors. Where effect estimates were not explicitly reported, risk differences (RDs) and corresponding 95% confidence intervals (CIs) were calculated from published event counts using the Newcombe–Wilson method without continuity correction [108,109]. Risk ratios (RRs) and 95% CIs were calculated using log-transformed standard errors where required. CIs crossing 0 (for RD) or 1 (for RR) were interpreted as indicating no statistically significant difference at a two-sided α level of 0.05.

Where trials reported clinical or microbiological failure rather than cure or eradication, corresponding cure or eradication proportions were calculated as the complement of failure to ensure consistency across studies.

For each outcome category, only studies reporting that specific outcome at comparable time points were included in the corresponding synthesis. Results were synthesized narratively by intervention type and outcome category.

Study characteristics and risk of bias assessments were summarized in tables, and outcome data were presented graphically to support structured narrative interpretation. Clinical and methodological heterogeneity across studies was explored qualitatively through comparison of intervention type, infection site, resistance profile, and outcome definitions.

Due to the limited number of included trials and the absence of quantitative meta-analysis, formal assessment of reporting bias (e.g., funnel plot analysis) was not performed.

A formal certainty-of-evidence assessment (e.g., using GRADE methodology) was not conducted due to the absence of quantitative pooling and substantial heterogeneity across included trials.

A generative artificial intelligence (GenAI) tool was used during manuscript preparation to assist with language refinement and structural organization. No AI tools were used for study selection, data extraction, statistical analysis, or interpretation of results. All outputs were reviewed, verified, and edited by the authors, who take full responsibility for the final content.

## 6. Conclusions

Across eight randomized controlled trials (RCTs) in critically ill ICU populations with carbapenem-resistant *A. baumannii* (CRAB), the combination regimens evaluated in randomised trials did not improve survival or overall clinical cure compared with monotherapy, despite occasional gains in microbiological clearance [54,55,56,57,58,59,60,61]. This lack of mortality benefit is biologically plausible: once sepsis triggers multi-organ dysfunction through dysregulated host responses, eliminating the pathogen rarely reverses established physiological injury [67,69].

By contrast, in non-sepsis infections where confounding from critical illness is absent, robust differences between antibiotics have been demonstrated. The ASPECT-cUTI trial, for example, showed significantly higher composite clinical and microbiological cure rates with ceftolozane–tazobactam versus levofloxacin in complicated urinary tract infections (cUTI) [75]. Such findings highlight settings where effective antibiotics can deliver measurable clinical benefit, providing a template for future CRAB research that focuses on non-sepsis indications, stratifies by infection source and resistance mechanism, and uses patient-centred outcomes.

A positive development is the emergence of pathogen-targeted agents such as sulbactam–durlobactam, which was non-inferior to colistin-based therapy and significantly less nephrotoxic in the phase 3 ATTACK trial [52,110]. Even without mortality superiority, its favourable safety profile represents an immediate clinical advance for severely ill patients.

In summary, current evidence does not justify routine combination therapy for sepsis due to drug-resistant *A. baumannii*. Management should prioritise early appropriate therapy, source control, and organ support. Future RCTs should avoid septic populations, focus on stable infections such as cUTI or skin/soft-tissue infections, prespecify composite clinical endpoints, and compare modern mechanism-matched therapies against best available monotherapy. Such trial designs are most likely to detect clinically meaningful treatment effects and translate them into practice.

Ultimately, in sepsis due to drug-resistant *A. baumannii*, host factors dominate outcomes, explaining the lack of survival benefit from combination therapy. The path to demonstrable efficacy lies in non-sepsis indications, where robust differences between antibiotics can be shown and safely leveraged in clinical care.

## Figures and Tables

**Table 1:** Characteristics of randomized controlled trials comparing antibiotic combination therapy versus monotherapy for drug-resistant *Acinetobacter baumannii* infections.

Study (Year)	Country/Setting	Design	Infection Types	Resistance Category	Treatment Arms	*n*
Aydemir et al., 2013 [56]	Turkey; single-centre ICU	Open-label RCT	CRAB VAP	CRAB	Colistin vs. Colistin + Rifampicin	43
Durante-Mangoni et al., 2013 [57]	Italy; multicentre ICUs	Open-label RCT (parallel)	HAP/VAP, BSI, cIAI	XDR *A. baumannii*	Colistin vs. Colistin + Rifampicin	210
Sirijatuphat et al., 2014 [59]	Thailand; single centre	Open-label RCT	CRAB infections	CRAB	Colistin vs. Colistin + Fosfomycin	94
Paul 2018 et al., (AIDA) [58]	Israel, Greece, Italy; 6 hospitals	Open-label RCT (blinded outcomes)	Severe infections (bacteraemia, VAP/HAP, urosepsis)	Carbapenem-non-susceptible GNB (77% *A. baumannii*)	Colistin vs. Colistin + Meropenem	406
Qin et al., 2018 [61]	China; single centre	RCT	VAP due to XDR *A. baumannii*	XDR *A. baumannii*	Tigecycline vs. Tigecycline + high-dose Cefoperazone–Sulbactam	42
Park et al., 2019 [54]	South Korea; single centre	RCT	Pneumonia due to colistin-resistant *A. baumannii*	Colistin-resistant *A. baumannii*	Colistin vs. Colistin + Rifampicin	9
Sirijatuphat et al., 2022 [60]	Thailand; single centre	RCT	CRAB infections	CRAB	Colistin vs. Colistin + Sitafloxacin	56
Kaye et al., 2022 (OVERCOME) [55]	International multicentre	Double-blind, placebo-controlled RCT	Pneumonia/BSI due to XDR GNB	XDR GNB incl. CRAB	Colistin + Meropenem vs. Colistin + Placebo	464

**Table 2:** Primary outcomes and adverse event profiles reported in randomized controlled trials comparing antibiotic combination therapy versus monotherapy for drug-resistant *Acinetobacter baumannii* infections.

Study (Year)	Primary Outcome	Key Findings	Adverse Events
Aydemir et al., 2013 [56]	Clinical/microbiological responses; VAP mortality	No difference; faster microbiological clearance with combination	Renal toxicity 23% overall; per-arm not reported
Durante-Mangoni et al., 2013 [57]	30-day all-cause mortality	No mortality difference; higher microbiological eradication with combination	Renal dysfunction ~26% overall; no per-arm difference
Sirijatuphat et al., 2014 [59]	28-day mortality; clinical & microbiological responses	Better microbiological response; trend to improved clinical outcomes	AKI 37.2% vs. 48.7%; abnormal LFTs similar
Paul 2018 et al., (AIDA) [58]	Day-14 clinical failure (composite)	No superiority of combination	Renal failure similar; fewer mild RIFLE-Risk events with combination
Qin et al., 2018 [61]	Clinical effectiveness; AEs	Higher clinical effectiveness with combination	Mild GI AEs similar; no renal impairment
Park et al., 2019 [54]	Day-14 responses; 30-day mortality	Higher mortality with combination (non-significant)	Not reported
Sirijatuphat et al., 2022 [60]	28-day mortality; clinical/microbiological responses; AEs	No difference in outcomes	AKI 53.8% vs. 45.8%
Kaye 2022 et al., (OVERCOME) [55]	28-day mortality	No difference in mortality or clinical failure	AKI similar; rare hypersensitivity/neurotoxicity

**Table 3:** Risk of bias assessment.

Study (Year)	Randomization Process	Deviations from Intended Interventions	Missing Outcome Data	Measurement of Outcome	Selection of Reported Result	Overall Risk of Bias
Aydemir et al., 2013 [56]	Some concerns	Some concerns	Low	Low	Low	Some concerns
Durante-Mangoni et al., 2013 [57]	Low	Some concerns	Low	Low	Low	Some concerns
Sirijatuphat et al., 2014 [59]	Some concerns	Some concerns	Low	Low	Low	Some concerns
Paul et al., 2018 (AIDA) [58]	Low	Some concerns	Low	Low	Low	Some concerns
Qin et al., 2018 [61]	Some concerns	Some concerns	Low	Low	Low	Some concerns
Park et al., 2019 [54]	Some concerns	Some concerns	Low	Low	Low	Some concerns
Sirijatuphat et al., 2022 [60]	Some concerns	Some concerns	Low	Low	Low	Some concerns
Kaye et al., 2022 (OVERCOME) [55]	Low	Low	Low	Low	Low	Low

**Table 4:** Inclusion and Exclusion Criteria.

Inclusion Criteria	Exclusion Criteria
Randomized controlled trials comparing combination antibiotic therapy versus monotherapy for the treatment of drug-resistant *Acinetobacter baumannii*.	Observational, preclinical, or non-randomized studies; case reports; reviews; conference abstracts; or studies without full-text availability.
Human subjects with confirmed MDR, XDR, or XDR-plus^+^ *A. baumannii* infections.	Publications not written in English.
Studies reporting clinical outcomes such as 28-day or 30-day all-cause or infection-related mortality.	Studies evaluating combination regimens involving non-antibiotic interventions.
Optional outcomes (only when clinical outcomes were reported): microbiological eradication, emergence of resistance during therapy, and adverse events or treatment-related toxicity.	Studies investigating monotherapy only.

## Data Availability

The data extracted and analysed during this review were derived from published randomized controlled trials cited within the manuscript. No new primary datasets were generated. Extracted summary data and analytic calculations are available from the corresponding author upon reasonable request.

## Supplementary Materials

The following supporting information can be downloaded at: https://www.mdpi.com/article/10.3390/antibiotics15040356/s1, Table S1: Full electronic search strategies used across databases; Table S2: PRISMA 2020 checklist detailing reporting of the systematic review.
