# Supplementary material for: The Role of Combination Antibiotic Therapy in Combatting Drug-Resistant Acinetobacter baumannii Infections: A Systematic Review of Randomised Control Trials
Source: Antibiotics (Basel). 2026 Mar 30;15(4):356. doi: 10.3390/antibiotics15040356 (PMC13112964; doi:10.3390/antibiotics15040356)
Supplement: Supplementary file 1 [file antibiotics-15-00356-s001.zip › Table S1-search_strategy.pdf]

Supplementary Table S1. Full electronic search strategies used across databases

Supplementary Table S1a. Ovid Medline Search strategy.

**1**	**(acinetobacter or baumannii).ti,ab.**
2	exp Acinetobacter baumannii/ or exp Acinetobacter/ or exp Acinetobacter Infections/
3	(resistan* or insensitive).ti,ab.
4	exp Drug Resistance, Multiple, Bacterial/ or exp Anti-Bacterial Agents/
5	(synerg* or adjuvant).ti,ab.
6	((combin* or "multi-drug") adj3 (treat* or therap*)).ti,ab.
7	exp Drug Therapy, Combination/
8	(antibiotic* or antimicrob*).ti,ab.
9	exp Anti-Bacterial Agents/
10	1 or 2
11	3 or 4
12	5 or 6 or 7
13	8 or 9
14	10 and 11 and 12 and 13
15	(randomized controlled trial or controlled clinical trial).pt. or randomized.ab. or placebo.ab. or randomly.ab. or trial.ab. or clinical trial as topic/
16	14 and 15
17	limit 16 to yr="2010-current"

Supplementary Table S1b. Ovid Embase search strategy.

**1**	**(acinetobacter or baumannii).ti,ab.**
2	exp Acinetobacter baumannii/ or exp Acinetobacter/ or exp Acinetobacter Infections/
3	(resistan* or insensitive).ti,ab.
4	exp Drug Resistance, Multiple, Bacterial/ or exp Anti-Bacterial Agents/
5	(synerg* or adjuvant).ti,ab.
6	((combin* or "multi-drug") adj3 (treat* or therap*)).ti,ab.
7	exp Drug Therapy, Combination/
8	(antibiotic* or antimicrob*).ti,ab.
9	exp Anti-Bacterial Agents/
10	1 or 2
11	3 or 4
12	5 or 6 or 7
13	8 or 9
14	10 and 11 and 12 and 13
15	exp randomized controlled trial/
16	controlled clinical trial/
17	random$.ti,ab.
18	randomization/
19	intermethod comparison/
20	placebo.ti,ab.
21	(compare or compared or comparison).ti,ab.
22	((evaluated or evaluate or evaluating or assessed or assess) and (compare or compared or comparing or comparison)).ab.
23	(open adj label).ti,ab.
24	((double or single or doubly or singly) adj (blind or blinded or blindly)).ti,ab.

25	double blind procedure/
26	parallel group*.ti,ab.
27	(crossover or cross over).ti,ab.
28	((assign$ or match or matched or allocation) adj5 (alternate or group* or intervention* or patient* or subject* or participant*)).ti,ab.
29	(assigned or allocated).ti,ab.
30	(controlled adj7 (study or design or trial)).ti,ab.
31	(volunteer or volunteers).ti,ab.
32	human experiment/
33	trial.ti.
34	or/15-33
35	(random$ adj sampl$ adj7 ("cross section$" or questionnaire$1 or survey$ or database$1)).ti,ab. not (comparative study/ or controlled study/ or randomi?ed controlled.ti,ab. or randomly assigned.ti,ab.)
36	cross-sectional study/ not (exp randomized controlled trial/ or controlled clinical trial/ or controlled study/ or randomi?ed controlled.ti,ab. or control group$1.ti,ab.)
37	(((case adj control$) and random$) not randomi?ed controlled).ti,ab.
38	(nonrandom$ not random$).ti,ab.
39	"random field$".ti,ab.
40	(random cluster adj3 sampl$).ti,ab.
41	(rat or rats or mouse or mice or swine or porcine or murine or sheep or lambs or pigs or piglets or rabbit or rabbits or cat or cats or dog or dogs or cattle or bovine or monkey or monkeys or trout or marmoset$1).ti. and animal experiment/
42	animal experiment/ not (human experiment/ or human/)
43	or/35-42
44	34 not 43
45	14 and 44
46	limit 45 to yr="2010-current"

Supplementary Table S1c. Ovid Global Health search strategy

1	(acinetobacter or baumannii).ti,ab.
2	exp Acinetobacter baumannii/ or exp Acinetobacter/ or exp Acinetobacter Infections/
3	(resistan* or insensitive).ti,ab.
4	exp Drug resistance/ or exp Drug resistance, bacterial/
5	(synerg* or adjuvant).ti,ab.
6	((combin* or "multi-drug") adj3 (treat* or therap*)).ti,ab.
7	exp Combination drug therapy/
8	(antibiotic* or antimicrob*).ti,ab.
9	exp Antibacterial agents/
10	1 or 2
11	3 or 4
12	5 or 6 or 7
13	8 or 9
14	10 and 11 and 12 and 13
15	(randomized controlled trial or controlled clinical trial or clinical trial or RCT or systematic review or meta-analys$).ti,ab.
16	(randomi?ed or randomly or trial or placebo or assign$ or allocat$ or double-blind or single-blind).ti,ab.
17	15 or 16
18	14 and 17
19	limit 18 to yr="2010-current"

Supplementary Table S1d. Cochrane CENTRAL search strategy

1	(acinetobacter OR "gram-negative bacteria"), ti.ab.
2	(resistan* OR "drug resistant"), ti.ab
3	(combination OR synerg* OR adjuvant OR "multi-drug") ti,ab.
4	(antibiotic* OR antimicrobial*) ti,ab.
5	1 and 2 and 3 and 4
6	limit 5 to yr="2010-current"
